# The genome of *Aeromonas salmonicida *subsp. *salmonicida *A449: insights into the evolution of a fish pathogen

**DOI:** 10.1186/1471-2164-9-427

**Published:** 2008-09-18

**Authors:** Michael E Reith, Rama K Singh, Bruce Curtis, Jessica M Boyd, Anne Bouevitch, Jennifer Kimball, Janet Munholland, Colleen Murphy, Darren Sarty, Jason Williams, John HE Nash, Stewart C Johnson, Laura L Brown

**Affiliations:** 1 NRC Institute for Marine Biosciences, 1411 Oxford Street, Halifax, NS, B3H 3Z1, Canada; 2 NRC Institute for Biological Sciences, 100 Sussex Drive, Ottawa, ON, K1A 0R6, Canada; 3 Present address: Biochemistry Department, Dalhousie University, Halifax, NS, B3H 1X5, Canada; 4 Present address: DNA Genotek Inc., 29 Camelot Drive, Ottawa, ON, K2G 5W6, Canada; 5 Present address: Public Health Agency of Canada, 130 Colonnade Road, Ottawa, ON, K1A 0K9, Canada; 6 Present address: DFO Pacific Biological Station, 3190 Hammond Bay Road, Nanaimo, BC, V9T 6N7, Canada

## Abstract

**Background:**

*Aeromonas salmonicida *subsp. *salmonicida *is a Gram-negative bacterium that is the causative agent of furunculosis, a bacterial septicaemia of salmonid fish. While other species of *Aeromonas *are opportunistic pathogens or are found in commensal or symbiotic relationships with animal hosts, *A. salmonicida *subsp. *salmonicida *causes disease in healthy fish. The genome sequence of *A. salmonicida *was determined to provide a better understanding of the virulence factors used by this pathogen to infect fish.

**Results:**

The nucleotide sequences of the *A. salmonicida *subsp. *salmonicida *A449 chromosome and two large plasmids are characterized. The chromosome is 4,702,402 bp and encodes 4388 genes, while the two large plasmids are 166,749 and 155,098 bp with 178 and 164 genes, respectively. Notable features are a large inversion in the chromosome and, in one of the large plasmids, the presence of a Tn*21 *composite transposon containing mercury resistance genes and an In2 integron encoding genes for resistance to streptomycin/spectinomycin, quaternary ammonia compounds, sulphonamides and chloramphenicol. A large number of genes encoding potential virulence factors were identified; however, many appear to be pseudogenes since they contain insertion sequences, frameshifts or in-frame stop codons. A total of 170 pseudogenes and 88 insertion sequences (of ten different types) are found in the *A. salmonicida *genome. Comparison with the *A. hydrophila *ATCC 7966^T ^genome reveals multiple large inversions in the chromosome as well as an approximately 9% difference in gene content indicating instances of single gene or operon loss or gain.

A limited number of the pseudogenes found in *A. salmonicida *A449 were investigated in other *Aeromonas *strains and species. While nearly all the pseudogenes tested are present in *A. salmonicida *subsp. *salmonicida *strains, only about 25% were found in other *A. salmonicida *subspecies and none were detected in other *Aeromonas *species.

**Conclusion:**

Relative to the *A. hydrophila *ATCC 7966^T ^genome, the *A. salmonicida *subsp. *salmonicida *genome has acquired multiple mobile genetic elements, undergone substantial rearrangement and developed a significant number of pseudogenes. These changes appear to be a consequence of adaptation to a specific host, salmonid fish, and provide insights into the mechanisms used by the bacterium for infection and avoidance of host defence systems.

## Background

The genus *Aeromonas *comprises a collection of Gram-negative bacteria that are widespread in aquatic environments and that have been implicated as causative agents of a number of human and animal diseases. *A. hydrophila*, *A. veronii *biovar sobria, *A. caviae*, *A. jandaei*, *A. veronii *biovar veronii, *A. schubertii *and *A. trota *have been associated with various human infections including gastroenteritis, wound infections and septicaemia [1]. *Aeromonas salmonicida*, a non-motile aeromonad, is the aetiological agent of a bacterial septicaemia in fish, called furunculosis [2-4]. Furunculosis is an important disease in wild and cultured stocks of salmonid and other fish species and can have significant negative economic impacts on aquaculture operations. Motile *Aeromonas *species have also been implicated as the causative agents of various fish septicemias [5]. *A. hydrophila *is also associated with red leg disease in amphibians and infections in turtles [6] and birds [7].

In addition to their role as disease agents, *Aeromonas *species can be found in non-pathogenic association with a variety of animals [8-10]. Most *Aeromonas *species are opportunistic pathogens, entering through wounds or affecting only stressed or otherwise immunocompromised hosts [1]. *A. salmonicida *subsp. *salmonicida*, however, is a specific pathogen of salmonid fish and is capable of causing disease in healthy fish at very low levels of infection (LD_50 _< 10 cfu by intraperitoneal injection [11]). Although Bergey's Manual of Systematic Bacteriology [12] recognizes five subspecies of *A. salmonicida*: *salmonicida*, *achromogenes*, *masoucida*, *smithia*, and *pectinolytica*, many laboratories currently classify *A. salmonicida *subsp. *salmonicida *as "typical" and any isolate deviating phenotypically as "atypical". Hosts for atypical strains include a wide variety of non-salmonid fish, as well as salmonids [4]. On the basis of DNA relatedness, *A. salmonicida *also includes a group of mesophilic, motile strains isolated from humans [12]. Morphological and biochemical differences such as pigment production, colony size and growth rate, haemolysis, and sucrose fermentation [4,13-15] are used to distinguish typical and atypical isolates. *A. salmonicida *subsp. *salmonicida *(i.e. typical) isolates grow well on blood agar with large colonies, produce a brown diffusible pigment, are haemolytic and do not ferment sucrose [12]. Historically, typical strains are thought to be extremely homogenous [16,17], and therefore any deviation in any of these characteristics has been considered enough evidence to classify a strain as "atypical" [13]. Phylogenetic analyses based on gene sequences [18,19] or biochemical analyses based on carbohydrates [20] appear to be better able to sort out the complex taxonomy and classification of *A. salmonicida *subspecies and related species.

*A. salmonicida *subsp. *salmonicida *appears to be an example of the evolution of pathogen specificity for a particular host from within a group of mainly opportunistic pathogens or commensal bacteria. It thus provides opportunities to identify genes involved in host invasion and virulence and to investigate the evolution of host specificity. In this communication, the genome, including both the chromosome and large plasmids, of an isolate of *A. salmonicida *subsp. *salmonicida *is characterized. The three small plasmids of this strain have been described previously [21]. Genes associated with virulence are identified and comparisons with the genome of *A. hydrophila *ATCC 7966^T ^[22] provide insights into the changes in the genome that may be associated with adaptation to fish hosts. The genome sequence is an essential tool for the understanding of the infection process of *A. salmonicida*.

## Results and discussion

### Genome features

**Figure 1:**
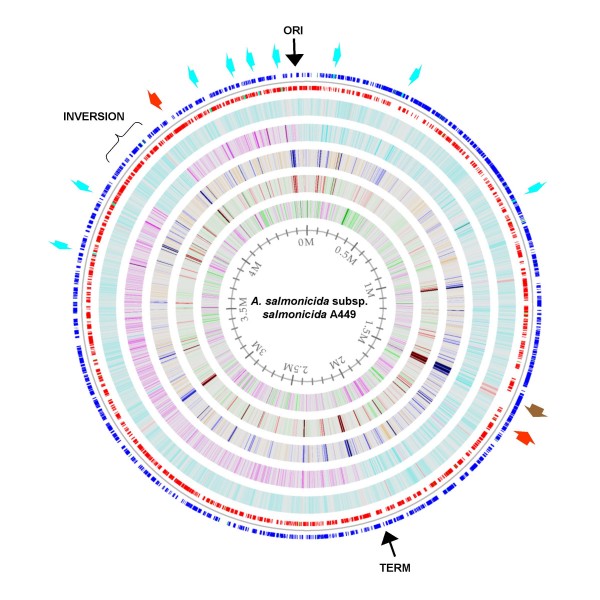
***A. salmonicida *subsp. *salmonicida *A449 chromosome**. A genome atlas representation of the *A. salmonicida *chromosome. Indicated outside the circular chromosome are the origin of replication (ORI), replication termination site (TERM), a large inversion in the genome (INVERSION), nine rRNA operons (light blue arrows), two prophages (red arrows) and the surface layer protein operons (brown arrow). Moving inward are circles representing annotations (blue: + strand CDS, red: – strand CDS, light blue: rRNA, green: tRNA), percent AT (blue: 20% to red: 80%), GC skew (pink: -0.10 to blue: 0.10), intrinsic curvature (yellow: 0.11 to blue: 0.21), stacking energy (green: -9.21 to red: -7.78) and position preference (green: 0.13 to pink: 0.16).

The genome of *A. salmonicida *subsp. *salmonicida *A449 (hereon A449) consists of a single circular chromosome, two large plasmids and three small plasmids. The 4,702,402 bp chromosome has a G+C content of 58.5% and contains 4388 genes, with 4086 encoding proteins (Table 1). Generally, the chromosome matches the restriction map previously constructed for this strain [23], although there are differences in the placement of some genes. The chromosome has a number of major structural features. The origin of replication (*oriC*), as inferred from the presence of multiple DnaA binding sites and GC skew, occurs at 4666400 – 4666750, which is approximately 35,700 bp from *dnaA *(Fig. 1). Replication terminates near 2134850 as judged by GC skew and the presence of a *dif*-like sequence, which has been recently implicated as the DNA replication terminus [24]. GC skew also detected the presence of a large inversion (3963279 – 4158772) that appears to have occurred between two identical insertion sequences. PCR analysis confirmed that this inversion was not due to misassembly of the sequence (not shown). In addition, two prophages have been detected by similarity to phage genes (Fig. 1, red arrows), but these regions of the chromosome do not show any obvious alteration in G+C content.

**Table 1:** *A. salmonicida *genome characteristics

	**chromosome**	**pAsa1**	**pAsa2**	**pAsa3**	**pAsa4**	**pAsa5**
length (bp)	4702402	5424	5247	5616	166749	155098
# genes	4388	9	9	10	178	164
# CDS	4086	8	7	9	173	154
# pseudogenes	155				5	10
# rRNA	28					
# tRNA	110					
# sRNA	9					
# misc. RNA		1	2	1		
# riboswitches	11					
# IS elements	71				7	10
# Tn*21*/In2					1	
# prophage	2					

Twenty-eight ribosomal RNA genes are encoded on the chromosome, arranged in nine operons, with one operon containing an extra copy of the 5S rRNA gene (Table 1; Fig. 1, light blue arrows). The nine operons are arranged around the origin of replication so as to be transcribed in the same direction as replication proceeds. Small variations (1 – 3 bp) in sequence occur between the copies of the rRNA genes, with only the "extra" 5S rRNA gene (rrfG1) having 6 bp that vary when compared to the other copies. A total of 110 tRNA genes are encoded on the A449 chromosome, most of which are present in at least two copies, and some of which occur in clusters of multiple tandem copies, similar to the *A. hydrophila *genome [22]. There are single genes for tryptophan (*trnW*) and selenocysteine tRNAs as well as a suppressor tRNA that translates TAG codons as tryptophan. This suppressor tRNA differs from the *trnW *sequence at only two bases, one of which is in the anticodon. Twenty-one protein coding genes appear to use the suppressor tRNA to allow the translation of the encoded protein. Analysis of the genome to identify small non-coding RNA features that regulate gene expression by binding to RNA or proteins [25] revealed the presence of nine small RNAs. In addition, 11 riboswitches, which regulate translation through the detection of small molecules [26], were detected near the 5' ends of genes they presumably regulate.

Two striking aspects of the A449 genome are the presence of large numbers of insertion sequences (IS) (n = 88) and pseudogenes (n = 170) on the chromosome and two large plasmids. Ten different types of IS are found in multiple copies in the A449 genome (Table 2) with ISAs7 present in 37 complete copies. One IS previously identified in *Aeromonas *species (ISAs4) [27] is not present in the A449 genome. In addition to the 88 complete IS elements, 14 partial IS sequences are present. This observation along with the finding that some IS are located within other IS, suggests that these dynamic elements have undergone recent transposition. Insertion sequences have also contributed to the apparent formation of pseudogenes, with more than 20 genes being interrupted by IS elements. Most pseudogenes, however, are created by small (1–37 bp) deletions or sequence duplications, although several genes have larger deletions. Additional pseudogenes appear to have arisen through mutations that introduce in-frame stop codons (TAA or TGA, but not TAG, due to the suppressor tRNA). These observations are in marked contrast to the *A. hydrophila *genome [22], which has no IS elements and only seven pseudogenes.

**Table 2:** A. salmonicida Insertion Elements

**Name**	**Length (bp)**	**# copies**	**IS family**	**Reference**
ISAs1	1223	2	ISAs1	[73]
ISAs2	1084	5	IS30	[73]
ISAs3	1326	4 (+2 partial)	IS256	Genbank NC_004338
ISAs4	1062	0	IS5	[27]
ISAs5	1233	12	IS3	[45]
ISAs6	1240	7 (+5 partial)	IS3	this work
ISAs7	1165	37 (+3 partial)	IS630	this work
ISAs8	754	3	IS1	this work
ISAs9	1624	4	IS3	this work
ISAs10	1229	2 (+1 partial)	IS30	this work
ISAs11	2614	12 (+3 partial)	IS21	this work

**Figure 2:**
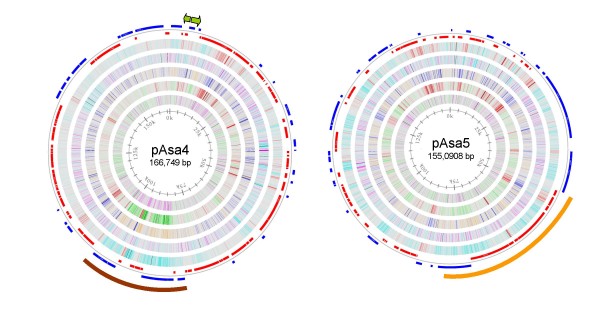
***A. salmonicida *subsp. *salmonicida *A449 large plasmids**. Genome atlas representations of the two *A. salmonicida *large plasmids. Green arrows outside the pAsa4 circle indicate tetracycline resistance genes and the brown bar indicates the position of the Tn*21 *containing the In2 integron. The orange bar on the pAsa5 circle indicates the position of the type III secretion apparatus operons. The interior circles are as described in Figure 1.

Both large plasmids contain genes involved in replication, plasmid partition and conjugative transfer. Plasmid 4 (pAsa4) carries an origin of replication that can be propagated in *E. coli*, since this plasmid was isolated by transformation of *E. coli *with a plasmid DNA extract from A449. This plasmid also contains a Tn*21 *composite transposon (bases 78182 – 101330) [28] that carries genes for resistance to mercury as well as an In2 integron encoding resistance to streptomycin/spectinomycin, quaternary ammonia compounds, sulphonamides and chloramphenicol (Fig. 2, brown bar). The Tn*21 *sequence has a considerably higher G+C content (61.43%) than the remainder of the plasmid (52.18%), as well as noticeable differences in stacking energy and position preference, as expected for a transposon.

### Virulence genes

#### Secretion systems

The most notable aspect of the other large plasmid, pAsa5, is the presence of genes for a type III secretion system (T3SS) (Fig. 2, orange bar; Table 3) that has been shown to be required for virulence in *A. salmonicida *[29,30]. The 36 genes encoding the T3SS needle apparatus and regulatory proteins are organized identically to those described previously for the partial sequence of *A. salmonicida *[31] and the complete T3SS sequences of *A. hydrophila*: AH-3 [32], AH-1 [33]and SSU [34]. As well, three presumptive effector proteins (AopH, AopO, Ati2) and their associated chaperones (SycH, SycO, Ati1) are located on pAsa5 (Table 3). Two of the effector proteins AopH and AopO (ASA_P5G009 and ASA_P5G098) show significant similarity to *Yersinia *YopH and YopO, and thus are expected to encode a protein tyrosine phosphatase and a protein serine/threonine kinase, respectively [30]. A third effector, Ati2 (ASA_P5G045), was identified by the presence of its chaperone (Ati1 (ASA_P5G046)) [35] and its similarity to hypothetical proteins in the T3SS operons of *Photorhabdus luminescens *and *Vibrio parahaemolyticus*. On the basis of conserved domain structure, this effector appears to have inositol polyphosphate phosphatase activity. A fourth, well-characterized effector, AexT (ASA_4266) [36,37], is encoded on the chromosome. Two pseudogenes also appear to encode T3SS effectors: ASA_0010 is located on the chromosome and is disrupted by an in-frame TAA stop codon; ASA_P5G084 is located on pAsa5 and is disrupted by a 20 bp duplication that results in a frameshift. Genes encoding presumptive T3SS chaperones are adjacent to both pseudogenes. The A449 genome does not include the AopP effector found in several *A. salmonicida *subsp. *salmonicida *strains including JF2267and ATCC 33658^T ^[38].

**Table 3:** Potential Virulence Genes of *Aeromonas salmonicida *A449

**Virulence ** **function**	**ASA Locus**	***A. hydrophila *ATCC 7966^T ^** **orthologue^1^**	**Description**	**Reference or Comments^2^**
Secretion	0514–0515	3785–3786	T2SS (ExeAB)	[55]
	3774–3785	0568–0579	T2SS (ExeC-N)	[56]
	P5G048 – 083	NP^3^	T3SS structural & regulatory proteins	[31]
	P5G008 & 009	NP	AopH effector & chaperone	[30]
	P5G097 & 098	NP	AopO effector & chaperone	[30]
	4266 & 4267	NP	AexT effector & chaperone	[36]
	P5G045 & 046	NP	Ati2 putative effector & chaperone	[35]
	P5G084 & 085	NP	putative T3SS effector & chaperone	frameshift
	0010 & 0011	NP	putative T3SS effector & chaperone	in frame TAA, frameshift
	2455 – 2470	1847 – 1832	T6SS operon	2455 & 2458 disrupted
	P4G080 – 082	1118 – 1119 1826 – 1827 1847 – 1848	T6SS	082 interrupted by Tn21 [39]
Adhesion	1422 – 1459	NP	surface layer & assoc. secretion system	[40,44]
	0346 – 0386	NP	Lateral flagella	0365, 0376 [45] disrupted
	1336 – 1360	1364 – 1388	Polar flagella	
	1484 – 1499	2847 – 2832	Polar flagella	1499 frameshift
	1505 – 1507	2826 – 2824	Polar flagella	1505 frameshift
	2656 – 2662	1698 – 1703	Polar flagella	2656 frameshift
	3725 – 3730	0519 – 0524	Type I pilus	
	0411 – 0414	3868 – 3671	Tap type IV pilus	0412 frameshift [93]
	2903 – 2915	1462 – 1450	Flp type IV pilus	2906, 2908 & 2913 disrupted [47]
	3938 – 3947	0383 – 0399	Msh type IV pilus	multiple gene deletion [47]
Toxins	3906	0438	aerolysin	[48]
	2854	1512	hemolysin	
	0826	3491	RTX toxin	
	2128	NP	cytolytic δ-endotoxin	
	2003	NP	zona occludens toxin	frameshift
	2015	NP	zona occludens toxin	IS insertion
Secreted enzymes	2540	2687	serine protease Ahe2	[51]
	3321	0978	zinc metalloprotease TagA	[52]
	3440	0851	elastase AhpB	
	1723	NP	metalloprotease	
	3723	0517	collagenase	
	1660	2713	AsaP1 protease	frameshift [53]
	0509	3791	glycerophospholipid cholesterol acyltransferase	[51]
	4288	0104	phospholipase A1	[54]
	0635	0635	phospholipase C	[54]
	1199	3126	extracellular nuclease	
	2206	2180	extracellular nuclease NucH	
	1286	1304	amylase Amy1	
	3455	0837	amylase AmyA	
	0873	3440	chitinase CdxA	
	2142	2363	chitinase Chi2	
	3320	0979	chitinase ChiB	
	0628	0628	pullulanase PulA	
Antibiotic resistance	P4G087 – 105	NP	Tn21/In2	
	P4G004 – 005	NP	tetracycline resist.	
	1191	3135	β-lactamase: ampC	[57]
	4346	4258	β- lactamase: ampS	[57]
	3612	0740	β- lactamase: cphA	[57]
Iron acquisition	1838 – 1851	2479 – 2473 1964 – 1970	amonabactin synthesis & uptake	[59,60]
	4368 – 4380	NP	anguibactin synthesis & uptake	[59,62]
	4363 – 4367	4275 – 4279	hydroxymate siderophore receptor	
	3328	0972	putative heme receptor	[59]
	3332 – 3336	0968 – 0964	heme uptake	[63]
Quorum sensing	3762	0556	N-acyl homoserine lactone synthase	[65]
	3763	0557	Quorum sensing regulon activator	[65]
	0697	0700	AI-2 synthase	
	2781	1576	Quorum sensing phosphorelay protein	
	3295	1004	Quorum sensing response regulator	

^1^indicates AHA_ number

^2^Disrupted genes are indicated

^3^NP – not present in *A. hydrophila *genome

Genes for a type VI secretion system (T6SS), which is also involved in the transfer of bacterial proteins into host cells, are encoded on the A449 chromosome (ASA_2455 – ASA_2470) (Table 3). These 16 proteins show high similarity to T6SS proteins from *A. hydrophila*, *P. aeruginosa *and other Gram-negative bacteria. Three additional genes usually associated with this operon are encoded on pAsa4 (ASA_P4G080 – ASA_P4G082). However, a key T6SS gene is interrupted in A449: the gene encoding IcmF (ASA_2458) contains a 5 bp deletion and is fused to the upstream coding sequence in the operon. In addition, two proteins transported by the T6SS are disrupted: a partial VgrG homolog is fused to a transposon subunit (ASA_2455), although a complete *vgrG *gene is encoded on pAsa4 (ASA_P4G080), and Hcp, which is encoded on pAsa4 (ASA_P4G082), is interrupted by an insertion sequence into which the Tn*21 *element has inserted. These gene disruptions are in contrast to *A. hydrophila *where the T6SS genes are uninterrupted and a functional T6SS has been demonstrated [39]. Since deletion of the *A. hydrophila icmF *homolog (*vasK*) blocks T6SS secretion [39], the A449 T6SS is unlikely to be functional. The presence of defects in two T3SS effectors and the T6SS suggests that A449 could be considerably more virulent, since functional versions of these genes would provide additional means to manipulate the response of the host.

A notable aspect of the T3SS and T6SS is the location of genes for these systems on both the chromosome and the large plasmids. For the T3SS, most of the genes are encoded on pAsa5, although one functional effector gene, *aexT*, and a putative effector pseudogene (ASA_0010), as well as their T3SS chaperone protein genes (ASA_4267, ASA_0011), are encoded on the chromosome. In *A. hydrophila *ATCC 7966T, T3SS genes are absent, while other *A. hydrophila *strains carry T3SS operons on the chromosome [32-34]. The ancestral state of the T3SS in the genus *Aeromonas *is thus unclear, making it difficult to surmise how it ended up in two locations in A449. The T6SS situation is somewhat reversed, with the majority of genes located on the chromosome, but with three genes located on pAsa4. Since *A. hydrophila *has a complete, intact T6SS on the chromosome, one might infer that these genes were transferred to pAsa4 following the acquisition of that plasmid, but prior to the capture of the Tn*21 *element.

#### Adhesins

Genes for several types of adhesins (e.g., surface layer, flagella, pili), which are important in host cell attachment and entry, are present in the A449 genome (Table 3). The abundant surface layer protein VapA (ASA_1438) [40], which has been implicated as an important virulence factor in several studies [41-43], is located downstream from an operon for a VapA-specific type II secretion system (ASA_1427 – ASA_1437). The identification of these genes as a VapA secretion system is based the observation that disruption of *spsE *(ASA_1427) blocks VapA secretion [44] and that many of the genes in this operon show some similarity to genes of the general secretion pathway (*exeA*-*N*). In the same region of the genome are multiple carbohydrate synthesis and modification genes (ASA_1422 – ASA_1426, ASA_1441 – ASA_1459) that appear to be involved in the synthesis of lipopolysaccharide, which anchors the surface layer to the cell. The genes involved in VapA synthesis and secretion have an unusually low G+C content that can be seen in Fig. 1 at approximately base 1500000 (Fig. 1, brown arrow).

Complete sets of genes for two types of flagella, lateral and polar, are also encoded in the A449 genome. The genes for lateral flagella are found in a single cluster (ASA_0346 – ASA_0386) but include two disrupted genes: *lafA*, encoding the lateral flagellin, which has been shown previously to be interrupted by an insertion sequence [45], and *lfgD*, encoding the lateral flagellar hook-capping protein, which has a 1 bp deletion. The genes for the polar flagella are dispersed around the genome in multiple operons (ASA_1336 – ASA_1360, ASA_1484 – ASA_1499, ASA_1505 – ASA_1507, ASA_2656 – ASA_2662), but also include interrupted genes: *flgL *(ASA_1499), encoding a flagellar hook-associated protein, has a 5 bp duplication; *flrA *(ASA_1505), encoding a transcriptional activator, contains a 13 bp deletion; and, *maf1 *(ASA_2656), encoding a motility accessory factor [46] has a 1 bp deletion. The disruption of genes involved in the production of both types of flagella suggests that neither structure can be synthesized, which is consistent with the characterization of *A. salmonicida *as non-motile.

An additional class of adhesins, the pili, is well-represented in the A449 genome with genes for four different pili (three type IV, one type I) distributed throughout the genome. The type I pilus operon (ASA_3725 – ASA_3730) appears to be complete and intact. However, for each of three types of type IV pili [47], there are frameshifted genes encoding proteins involved in pilin assembly (*tap*, *flp*) or a multiple gene deletion (*msh*) (Table 3). Nevertheless, a mutant deleted for *tapA *showed reduced virulence when delivered by immersion, but not by intraperitoneal injection, suggesting a role for the Tap pilus in host invasion [47].

#### Toxins

Another class of putative virulence factors are pore-forming toxins that create channels in host membranes resulting in cell lysis (Table 3). Aerolysin, one of the earliest virulence factors to be discovered among *Aeromonas *spp. [48], is represented by two genes in the A449 genome: ASA_3906, which encodes the classical aerolysin [48], and ASA_2854, which encodes a conserved hemolysin found in other *Aeromonas *species, *Vibrio *species and *Listonella anguillarum*. A large (9588 bp) gene, *asx *(ASA_0826), encodes an RTX (repeats in toxin) protein, homologs of which are important virulence determinants in a range of Gram-negative bacteria [49]. The A449 genome also encodes a cytolytic delta-endotoxin (ASA_2128) that is 61% similar to the *Bacillus thuringiensis *insecticidal toxin CryET29 (Genbank accession AAK50455), which may be an indication of interaction with invertebrates. Two additional genes (ASA_2003 and ASA_2015) encode proteins that are 53 and 44% similar to the zonula occludens toxin (Zot) of *Colwellia psychrerythraea *(Genbank accession YP_267119). Zot was first described in *Vibrio cholerae *as a toxin that transiently loosens intracellular tight junctions in the intestinal mucosa [50]. In *V. cholerae*, Zot is associated with cholera toxin A and B and is also encoded on the CTX*Φ *plasmid [50]. In A449, however, both genes are interrupted, either by an IS (ASA_2015) or by a single bp insertion (ASA_2003), suggesting that functional proteins can not be synthesized from them.

#### Secreted enzymes

An additional class of potential virulence factors in *A. salmonicida *are extracellular enzymes, some of which have been previously investigated (Table 3). Among secreted proteases are a serine protease previously tested for its contribution to virulence (ASA_2540) [51], a zinc metalloprotease (TagA, ASA_3321) implicated in complement inhibition [52], another secreted metalloprotease (ASA_1723) and a microbial collagenase (ASA_3723). A gene encoding an extracellular endopeptidase (AsaP1, ASA_1660) contributing to virulence in atypical *A. salmonicida *strains [53] is present, but interrupted by a single bp insertion. The phospholipases encoded by *satA *(glycerophospholipid cholesterol acyltransferase, ASA_0509), *pla *(phospholipase A1, ASA_4288) and *plc *(phospholipase C, ASA_0635) have been investigated for their role in virulence in *A. salmonicida *[51] and *A. hydrophila *[54]. Extracellular nucleases (ASA_1199, ASA_2206), amylases (ASA_1286, ASA_3455), pullulanase (ASA_0628) and chitinases (ASA_0873, ASA_2142, ASA_3320) may also contribute to A449 virulence. All of these enzymes have a predicted Sec-dependent signal sequence and are expected to be secreted via the type II secretion system (*exeA-N*, ASA_0514–0515, ASA_3777–3785) [55,56].

#### Antibiotic resistance

In addition to the antibiotic resistance genes encoded in the Tn*21 *element, pAsa4 also carries genes for tetracycline resistance: *tetA*(E) (ASA_P4G005) encodes a class E tetracycline efflux pump that is presumably regulated by the adjacent class E tetracycline repressor protein (*tetR*(E), ASA_P4G005). Three β-lactamase genes, *ampC *(ASA_1191), *ampS *(ASA_4346) and *cphA *(ASA_3612), previously described in *A. sobria *(as *cepS*, *ampS *and *imiS*, respectively) [57] are carried on the A449 chromosome (Table 3). The presence of more than 25 genes for multidrug resistance and major facilitator efflux family proteins indicates that A449 carries an array of genes to counteract antimicrobials.

#### Iron acquisition

Iron acquisition is an important virulence factor for many bacterial pathogens and for *A. salmonicida*, it may also be a key process for survival in aquatic environments. Mesophilic *Aeromonas *species have been found to produce two types of catecholate siderophores, amonabactin and enterobactin [58]. When A449 is grown under low iron conditions, either *in vivo *or in the presence of 2,2'-dipyridyl, three outer membrane proteins are induced that appear to be ferric siderophore or heme receptors [59]. On the A449 chromosome, both of the ferric siderophore receptors are located adjacent to clusters of genes encoding ABC-type ferric transporter subunits as well as non-ribosomal peptide synthetase modules, suggesting complete systems for siderophore synthesis and uptake. The gene for the FstC receptor is located within a cluster (ASA_1838 – ASA_1851) that includes the amonabactin synthesis gene [60], indicating that these genes are likely involved in the synthesis and uptake of amonabactin. The gene for the FstB receptor is encoded in a gene cluster (ASA_4368 – ASA_4380) that is similar to the *Listonella anguillarum *anguibactin and the *Acinetobacter baumannii *acinetobactin synthesis genes [61], suggesting that A449 has the ability to synthesize and recapture an anguibactin-like siderophore. Some of the genes in this cluster have been recently characterized in *A. salmonicida *and shown to be required for siderophore synthesis [62]. Adjacent to this gene cluster are five genes (ASA_4363 – ASA_4367) encoding a hydroxamate-type ferric siderophore receptor and an ABC transporter system, indicating that A449 may also use a hydroxamate siderophore for iron acquisition. The gene for a presumptive heme receptor, *hupA *(ASA_3328), that is induced by low iron conditions [59], is located near *hutZXBCD *(ASA_3332 – ASA_ 3336), which encode proteins involved in heme uptake and utilization [63]. Genes for several additional Ton-B dependent outer membrane receptors that may be involved in heme or hemoprotein transport are also present in the A449 genome, but require further characterization to establish their function.

#### Quorum sensing

Another bacterial process implicated in virulence is quorum sensing [64]. The A449 chromosome contains the *luxI *and *luxR *homologs, *asaI *(ASA_3762) and *asaR *(ASA_3763), which encode proteins for the synthesis of the acylhomoserine lactone quorum sensing molecule and the transcriptional regulator that responds to it, respectively [65]. In addition, genes for a second quorum sensing pathway that uses autoinducer-2 [66] are present: *luxS *(ASA_0697) encodes the autoinducer-2 synthase, *luxU *(ASA_2781) is a putative phosphorelay protein involved in transduction of the signal and *luxO *(ASA_3295) is a transcriptional response regulator (Table 3). Other unidentified genes in the A449 genome may also participate in these systems since in *Vibrio *spp. receptor proteins and multiple small RNAs are involved in the complete signal transduction pathway [67].

### Comparison to the *A. hydrophila *genome

**Figure 3:**
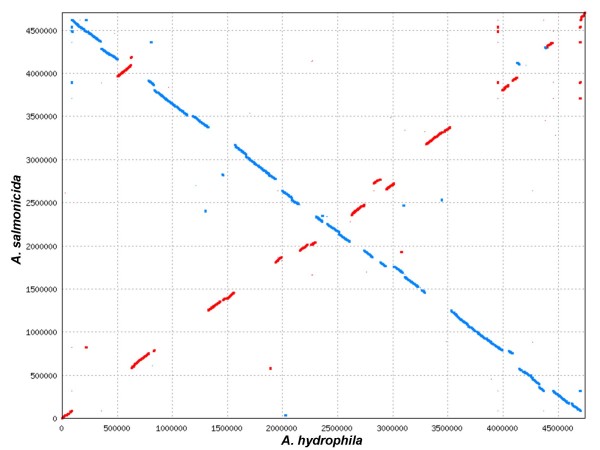
**Comparison of *A. salmonicida *and *A. hydrophila *chromosomes**. Mummer comparison of the *A. salmonicida *and *A. hydrophila *chromosomes. Red lines/dots indicate forward sequence matches. Blue lines/dots indicate reverse matches.

The genome sequence of *A. hydrophila *ATCC 7966^T ^[22] provides an excellent basis for comparative sequence analysis leading to enhanced understanding of genome evolution within the genus *Aeromonas*. A comparative analysis of the two chromosomes using Mummer [68] is shown in Fig. 3. Due to an inversion around the origin of replication, the A449 sequence primarily aligns with the *A. hydrophila *sequence on the reverse strand (blue line in Fig. 3). As expected for two chromosomes of nearly the same size, there are no large gap regions indicative of significant insertions or deletions. Nearly all regions of sequence similarity fall along one of the diagonals, indicating generally similar gene and sequence order. Approximately 15 large sequence inversions (red lines in Fig. 3) around the origin of replication have occurred in the A449 chromosome relative to the *A. hydrophila *chromosome, accounting for the regions of forward strand alignment. The large inversion already noted in the A449 chromosome, which appears to be an evolutionarily recent change since it is bounded by transposons and is absent in *A. hydrophila*, stands out as a red line along the blue (reverse strand) diagonal at 500,000 bp in the *A. hydrophila *sequence.

On a global scale, the A449 and *A. hydrophila *chromosomes appear generally similar and encode similar numbers of proteins (4086 in A449, 4128 in *A. hydrophila*). However, there are multiple instances of single gene or operon loss and gain between the two genomes, leading to a 9% difference in gene content. There are 477 coding sequences (CDS) present in the *A. salmonicida *chromosome that are not found on the *A. hydrophila *chromosome. Many of these are transposon (101 CDS) or phage related (69 CDS) and 122 represent CDS unique to *A. salmonicida*. However, there are also 97 conserved hypothetical CDS found in other bacterial species and 88 known CDS that are present in the *A. salmonicida *genome, but not in that of *A. hydrophila*. Conversely, the *A. hydrophila *genome contains 278 CDS not present in *A. salmonicida *(72 unique CDS, 67 conserved hypothetical CDS and 139 known CDS). Clearly, significant changes in gene content have occurred following the separation of these two species.

### Pseudogenes

An additional obvious difference between the *A. salmonicida *and *A. hydrophila *genomes is the number of pseudogenes present. The *A. hydrophila *genome [22] has only 7 pseudogenes: 2 in tRNA genes, 2 protein CDS with in-frame stop codons and 3 frameshifted protein CDS. Only one of these CDS (AHA_2264) is present in *A. salmonicida *(ASA_2042) and both genes contain the same frameshift. To investigate the frequency and occurrence of frameshifts in the genus *Aeromonas*, we attempted to amplify and sequence 16 *A. salmonicida *pseudogenes (Additional file 1) having a variety of lesions from five *A. salmonicida *strains (two strains of subspecies *salmonicida*, one each of subspecies *masoucida*, *achromogenes *and *smithia*) and from one strain each of five other *Aeromonas *species (*hydrophila*, *veronii*, *caviae*, *sobria *and *bestiarum*) (Table 4). In addition, these sequences were amplified from A449 cDNA to determine whether transcriptional frameshifting corrected any of them. All the cDNA sequences were identical to the genomic sequence (Table 4 and Additional file 1). While most of the genes could be amplified from the *A. salmonicida *strains and subspecies, the amplification of genes from the other *Aeromonas *species was considerably less successful (Table 4 and Additional file 1), presumably due to sequence changes at the primer sites. However, it is clear (Table 4) that in the single species of *A. salmonicida *subsp. *masoucida*, *achromogenes *and *smithia *that were tested, only 3 or 4 of the pseudogenes are present and that none of the amplified sequences from the other *Aeromonas *species showed disruptions. While this analysis tests less than 10% of the A449 pseudogenes and although the data are incomplete for many of the non-*A. salmonicida *species, these pseudogenes appear to be limited to *A. salmonicida *with the majority present only in *A. salmonicida *subsp. *salmonicida*.

**Table 4:** Summary of disrupted genes in *Aeromonas *species

**Species**	**# genes amplified^1^**	**# disrupted genes**	**% disrupted**
A449	16^2^	16	100
A449 cDNA	16	16	100
*A. salmonicida *subsp. *salmonicida *ATCC 33658^T^	15	15	100
*A. salmonicida *subsp. *salmonicida *ATCC 51413 (non-pigmented)	16	16	100
*A. salmonicida *subsp. *masoucida *ATCC 27013^T^	13	3	23
*A. salmonicida *subsp. *achromogenes *ATCC 33659^T^	14	3	21
*A. salmonicida *subsp. *smithia *ATCC 49393^T^	15	4	27
*A. bestarium *ATCC 51108^T^	7	0	0
*A. veronii *bv. sobria ATCC 9071	4	0	0
*A. sobria *ATCC 43979^T^	2	0	0
*A. caviae *ATCC 15468^T^	2	0	0
*A. hydrophila *ATCC 7966^T^	13^2^	0	0

^1 ^The genes tested are described Additional file 1. Primers used for amplification are described in Additional file 2.

^2^Sequences used were from the complete genomes.

### Genomic evidence for pathogen speciation

Analyses of the genomes of bacterial pathogens provide evidence that three key processes, the acquisition of mobile genetic elements, genome rearrangements and gene loss in the process of adapting to a specific host, result in substantial changes in the genomes of pathogens (see [69] for a recent review). Since its separation from the last common ancestor with *A. hydrophila*, A449 appears to have acquired multiple plasmids, two prophages, a variety of IS elements and a number of individual genes and operons, presumably through horizontal gene transfer. While mechanisms for the acquisition of prophage, plasmids and insertion sequences are understood, mechanisms for the gain of individual genes, such as the *B. thuringiensis *toxin, or operons, such as VapA and its secretion system, are less obvious. The acquisition of foreign DNA appears to be an ongoing process in *A. salmonicida *based on the diversity of plasmids identified in various strains [70-72]. IS transposition also continues to be active in A449 since mutants can be generated by IS transposition ([73], JMB unpublished results), usually by growth under stressful conditions such as elevated temperature (30°C).

Substantial genetic rearrangements have occurred in the A449 genome, relative to the *A. hydrophila *genome. Many of these rearrangements have been assisted by the presence of IS elements, such as the apparent transfer of T6SS genes to pAsa4 and the large inversion. Most of the other inversions, relative to *A. hydrophila*, do not appear to be associated with IS elements, indicating that other mechanisms are also generating genetic rearrangements. While these large scale rearrangements do not obviously affect gene sequences, reorientation of large regions relative to the origin of replication may impact the regulation of gene expression.

The third trait of recently evolved pathogen genomes, gene loss or decay, has also occurred frequently in the A449 genome. The number of A449 pseudogenes, 170, is comparable to that seen in *Yersinia pestis *(~150) [74], but less than other recently evolved human pathogens such as *Salmonella enterica *serovar Typhi CT18 (>200) [75] or *Mycobacterium leprae *(>1100) [76]. Several of the A449 pseudogenes prevent the expression of cell surface structures such as flagella and pili. Loss of flagellar motility is common among recently emerged pathogens [69], perhaps as a means to evade the host innate immune system. Loss of genes for type IV pili is also associated with pathogen speciation [77] and may also help pathogens avoid innate immune responses [78]. Significant accumulations of pseudogenes are also found among genes for transcriptional regulators (17 pseudogenes), genes encoding carbohydrate synthesis and modification enzymes (12 pseudogenes) and genes for basic metabolic enzymes (e.g., sulfite reductase, α and β galactosidase, acetolactate synthase, etc.) (10 pseudogenes). Compared to *A. hydrophila *ATCC 7966^T^, the "jack of all trades" [22], the accumulation of pseudogenes in A449 has considerably reduced its capacity to produce some organelles (e.g., pili or flagella) and to synthesize some enzymes and their products.

The A449 genome thus carries all the hallmarks of an organism that has undergone adaptation to a specific host. Clearly, substantial horizontal gene transfer, genome rearrangements and gene decay have occurred in A449 relative to *A. hydrophila *ATCC 7966^T^. The small survey of pseudogenes in other members of the genus *Aeromonas *suggests that pseudogene accumulation coincided with the speciation of *A. salmonicida *but increased substantially during the evolution of the subspecies *salmonicida*. Further analysis of *Aeromonas *sequences and genomes should provide insights into the process and timing of the evolution of host specialization as well as a better understanding of the genes and proteins involved in virulence.

## Conclusion

The genome of *A. salmonicida *subsp. *salmonicida *A449 consists of a circular chromosome and five plasmids that encode more than 4700 genes. A large number of genes encoding potential virulence factors have been identified, although a number of them have been disrupted to become pseudogenes. The acquisition of plasmids, insertion sequences and pseudogenes, along with large genome rearrangements is indicative of a genome that has decayed to adapt to the environment of a specific host.

## Methods

### Bacterial Strains

*Aeromonas salmonicida *subsp. *salmonicida *A449 was originally isolated from a brown trout in the Eure river, France by Christian Michel in 1975 [79]. Other *Aeromonas *strains were obtained from the American Type Culture Collection (ATCC): *A. salmonicida *subsp. *salmonicida *ATCC 33658^T^, *A. salmonicida *subsp. *salmonicida *ATCC 51413 (non-pigmented), *A. salmonicida *subsp. *masoucida *ATCC 27013^T^, *A. salmonicida *subsp. *achromogenes *ATCC 33659^T^, *A. salmonicida *subsp. *smithia *ATCC 49393^T^, *A. bestarium *ATCC 51108^T^, *A. veronii *bv. sobria ATCC 9071, *A. sobria *ATCC 43979^T^, *A. caviae *ATCC 15468^T ^and *A. hydrophila *ATCC 7966^T^.

### Genome Sequencing

A mixed strategy was employed for sequencing the genome of A449. A shotgun library was generated by cloning hydro-sheared and end-repaired 1–2 kb genomic inserts into the plasmid vector pUC19. Clones from this library were sequenced [80] from both direction on Li-Cor 4200 and MegaBace 1000 instruments. As well, a BAC library was constructed by partial digestion of genomic DNA with EcoRI and cloning in pBACe3.6 [81]. Twelve clones from this library were sequenced completely. All reads were assembled in gap4 [82] to produce ~2100 contigs with approximately 6× coverage. Contigs were joined using a read-pair approach as well as a two-step PCR-based approach involving two primers at the contig ends and a random primer. For contig closure, a fosmid library was made in the EpiFOS vector (Epicentre Biotechnologies) and clones were end-sequenced to locate their position in the assembly. Sequence from these clones was used for confirming assembly as well as to fill the remaining gaps. Finally, the sequence was completely disambiguated and polished by sequencing genomic PCR products generated with flanking primer pairs. Presumptive plasmid contigs were identified by similarity to common plasmid encoded genes, removed from the main assembly and joined by PCR experiments using primers at the contig ends. pAsa4 was cloned into *E. coli *DH5α by transformation with a plasmid DNA preparation from A449 and selection on chloramphenicol. This clone was used to identify pAsa4 contigs and to join and polish the sequence. The A449 chromosome and plasmid 4 and 5 sequences have been deposited in Genbank (NC_009348, NC_009439, NC_009350).

### Annotation

Initial analysis of the genome sequences was done using a script written in Perl and relying heavily on the BioPerl modules [83]. The script initially searched for rRNA and transposon sequences using Blastn [84] followed by a tRNA search using tRNAscan-SE [85]. sRNA sequences were also identified with rfam_scan.pl which uses Blast and INFERNAL [86] searches of the Rfam database [87]. Open reading frames were identified with Glimmer2 [88] and searched for similarity using Blastp and for conserved domains with CDD [89]. Sequences between open reading frames with Blastp or CDD hits were extracted and further searched with Blastx and Blastn. All search results were assembled in an EMBL feature table file for editing in Artemis [90]. Final annotation was done by hand in Artemis. Chromosome and large plasmid representations were produced using the Genome Atlas website [91]. Comparisons between the *A. salmonicida *and *A. hydrophila *chromosomes used the Mummer package [68].

### Frameshift Analysis

To investigate the presence of frameshifts in other *Aeromonas *species and subspecies, primers flanking frameshift sites (Additional file 2) were designed with Primer 3 [92]. *Aeromonas *species and subspecies were grown in tryptic soy broth and DNA was extracted for use as the template in standard PCR reactions. PCR products were gel purified and sequenced directly. Unsuccessful amplifications were attempted at least two more times using a lower annealing temperature. RNA extraction and cDNA synthesis were as described previously [35]. Sequences were deposited in Genbank under accession numbers FJ178190–FJ178298.

## Authors' contributions

MER coordinated the project, edited and assembled sequence, annotated the sequence, conceived of the frameshift experiments, carried out the A. hydrophila genome comparison and wrote and edited the manuscript. RKS made the shotgun library, directed the DNA sequencing, designed and directed joining approaches and polished the sequence. BC edited and assembled the sequence and contributed bioinformatic analyses. JMB cloned pAsa4, contributed to annotation and edited the manuscript. AB performed and analyzed joining experiments. JK designed and implemented software for tracking clones and sequences. JM constructed and characterized the BAC library. CM performed and analyzed assembly PCRs and contributed to plasmid sequences. DS designed, performed and analyzed the frameshift PCR experiments. JW characterized and analyzed BAC clones and sequences. JHEN designed and directed joining approaches. SCJ and LLB co-led the conception and design of the host-pathogen interaction program and contributed to the writing and editing of the manuscript. All authors read and approved the final manuscript.

## Supplementary Material

Additional file 1 Additional Table 1. Details of the analysis of disrupted genes in *Aeromonas *species. Click here for file

Additional file 2 Additional Table 2. Primers for pseudogene amplification. Click here for file
