# Supplementary material for: The genome of Aeromonas salmonicida subsp. salmonicida A449: insights into the evolution of a fish pathogen
Source: BMC Genomics. 2008 Sep 18;9:427. doi: 10.1186/1471-2164-9-427 (PMC2556355; doi:10.1186/1471-2164-9-427)
Supplement: Additional file 1 — Additional Table 1. Details of the analysis of disrupted genes in Aeromonas species. [file 1471-2164-9-427-S1.doc]

## Additional Table 1 - Details of the analysis of disrupted genes in *Aeromonas* species

| ASA  locus | Gene | Pseudogene Sequence | A449 | A449  cDNA | *A. salmonicida* subsp. | | | | | *A. bes-tiarum* | *A. veronii* | *A. sobria* | *A. caviae* | *A. hy-drophila* |
| --- | --- | --- | --- | --- | --- | --- | --- | --- | --- | --- | --- | --- | --- | --- |
|  |  |  |  |  | sal | sal(np) | masou | achrom | smithia |  |  |  |  |  |
| 0213 | *lacZ* | A ins. | + | + | + | + | - | - | - |  |  |  |  | - |
| 0412 | *tapC* | T del. | + | + | + | + | + | + | + | - | - | - | - | - |
| 0613 | NGD | 37 bp^1^ ins. | + | + | + | + | - | - | - |  | - |  |  | NP |
| 0938 | *cysJ* | 11 bp^2^ del. | + | + | + | + | - | - | - | - | - |  |  | - |
| 1019 | *fumC* | TAG^3^ | + | + | + | + | + | + | + | - |  |  |  | - |
| 1621 | NGD | TAA | + | + | + | + | - | - | - |  |  |  |  | - |
| 1660 | *asaP1* | G ins. | + | + | + | + | + | +^7^ | + | - |  |  |  | - |
| 2601 | *tapF* | 7 bp^4^ ins. | + | + | + | + | - | - | - | - |  | - | - | - |
| 2906 | *flpI* | CTGT ins. | + | + | + | + | -^8^ | - | - |  |  |  |  | - |
| 2908 | *flpG* | TAA | + | + | + | + | - | - | - |  |  |  |  | - |
| 2913 | *flpB* | TAA | + | + | + | + | - | - | - |  |  |  |  | - |
| 3210 | *ilvB* | 13 bp^5^ del. | + | + | + | + |  | - | - | - |  |  |  | - |
| 3278 | NGD | A ins. | + | + | + | + |  |  | + | - |  |  |  | - |
| 3440 | *ahpB* | C del. | + | + | + | + | - | - | - |  |  |  |  | - |
| P5G014 | NGD | A ins. | + | + | + | + | - |  |  |  |  |  |  | NP |
| P5G084 | *aopX* | 20 bp^6^ ins. | + | + |  | + |  | - | - |  | - |  |  | NP |

Species used are described in Table 4 and PCR primers are described in Additional Table 2. Sequences for A449 and *A. hydrophila* are from the genome sequences.

+ = pseudogene sequence present; - = pseudogene sequence absent; ins. = insertion; del. = deletion; NGD = no gene designation; NP = gene not present; blank cells = no PCR product

^1^TATGGTGCCTTCAACCAGACGGTGCTGAACCTGGCCC

^2^GGTGGGGGAAG

^3^TAG is translated as tryptophan in A449 due to a suppressor tRNA

^4^TCAGCGC

^5^TCTCCCCAAGCAG

^6^GCAAAGCGCCCCCGATAGCG

^7^*A. salmonicida* subsp. *achromogenes* has a compensatory, second-site deletion (CTGCCGGGGA) to restore function of this gene

^8^*A. salmonicida* subsp. *masoucida* has a 13 bp deletion (ATGATGGTCTGAG) that disrupts this gene
