# Supplementary material for: The genome of Aeromonas salmonicida subsp. salmonicida A449: insights into the evolution of a fish pathogen
Source: BMC Genomics. 2008 Sep 18;9:427. doi: 10.1186/1471-2164-9-427 (PMC2556355; doi:10.1186/1471-2164-9-427)
Supplement: Additional file 2 — Additional Table 2. Primers for pseudogene amplification. [file 1471-2164-9-427-S2.doc]

## Additional Table 2. Primers for pseudogene amplification

| **ASA_** | **Gene** | **5’ primer** | **3’ primer** |
| --- | --- | --- | --- |
| 0213 | *lacZ* | GAGAACGGGGTTGTCGTG | GTCCAGTTGCCACTCGCT |
| 0412 | *tapC* | CCTTTCCACCACCTTCTC | agagccatggcaagccgt |
| 0613 | NGD^1^ | TCCCACTACGGCTTGCTC | CCCCATGATCTCGTCCAG |
| 0938 | *cysJ* | GGGTCTGGTTCGACAACG | ATCCACCACTTGGGCAGA |
| 1019 | *fumC* | CAACAAATTCGAGGCGCT | GCAGACGCACGCTCTGTA |
| 1621 | NGD | CAGTGGGGTTGGCTATCG | CTGCAAGGCGGCACTATT |
| 1660 | *asaP1* | GATGGTCAACCGGTCAGC | GGTGATGGCGTTGGACTC |
| 2601 | *tapF* | tttcataggtatcggcaatcc | GGCTTCACCGTCATCGTC |
| 2906 | *flpI* | gacaagaagcaggcggcg | tgatcaccagctctatgc |
| 2908 | *flpG* | GTTTTATCGCCCATCCCC | AGCAGTGCCAGCCAGAAC |
| 2913 | *flpB* | GCCCGTCACAGTGCCTTA | TTCCCTTGAGTGCCTTGG |
| 3210 | *ilvB* | CTGCACATTGATGCCCTG | TGTCTGCTGACCGTTGGA |
| 3278 | NGD | GGATTATCCCACCCTGCC | CAGCATGGCGCTCTTTTT |
| 3440 | *ahpB* | CTCGGTGGGCAGATAAGC | GAAGTCCGCGCCATAGAA |
| P5G014 | NGD | CGGACACGCCTTTGTTTC | TTCAACGCATCAACGGAA |
| P5G084 | *aopX* | GCTGTTCGAGCAATCCGT | CCAGCGCCTGTTTCAGTT |

^1^NGD = no gene designation
